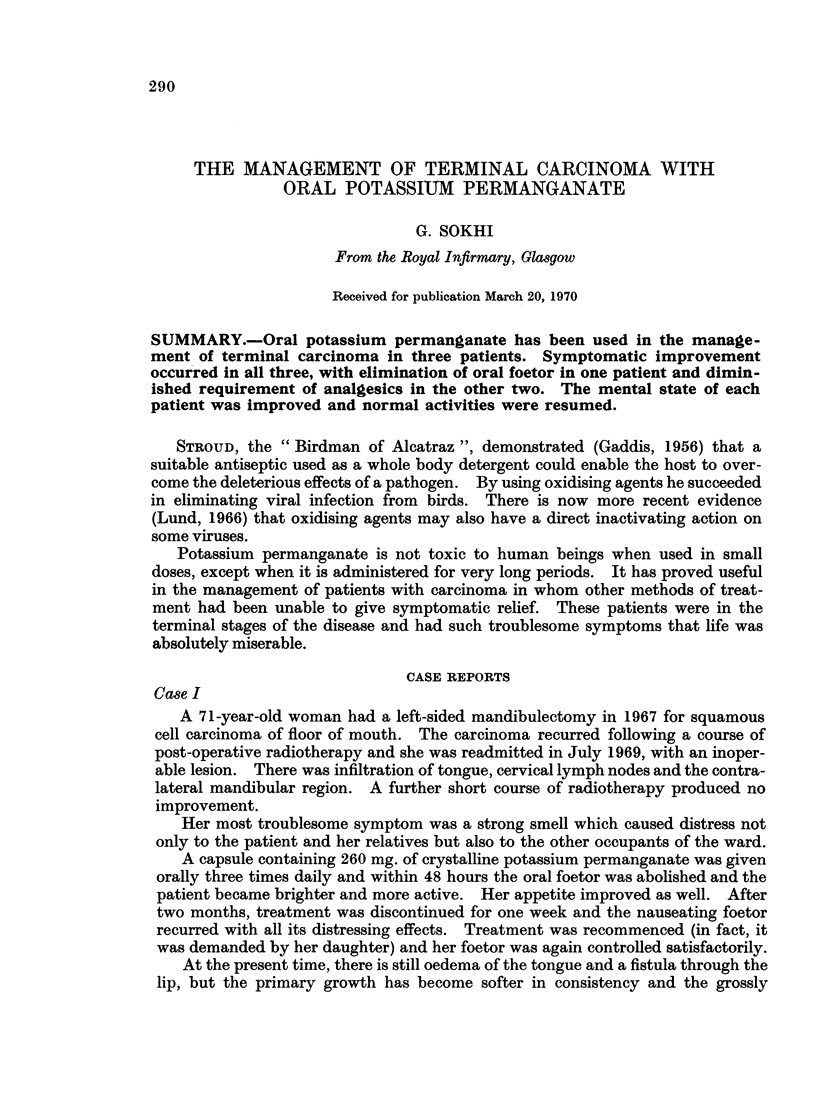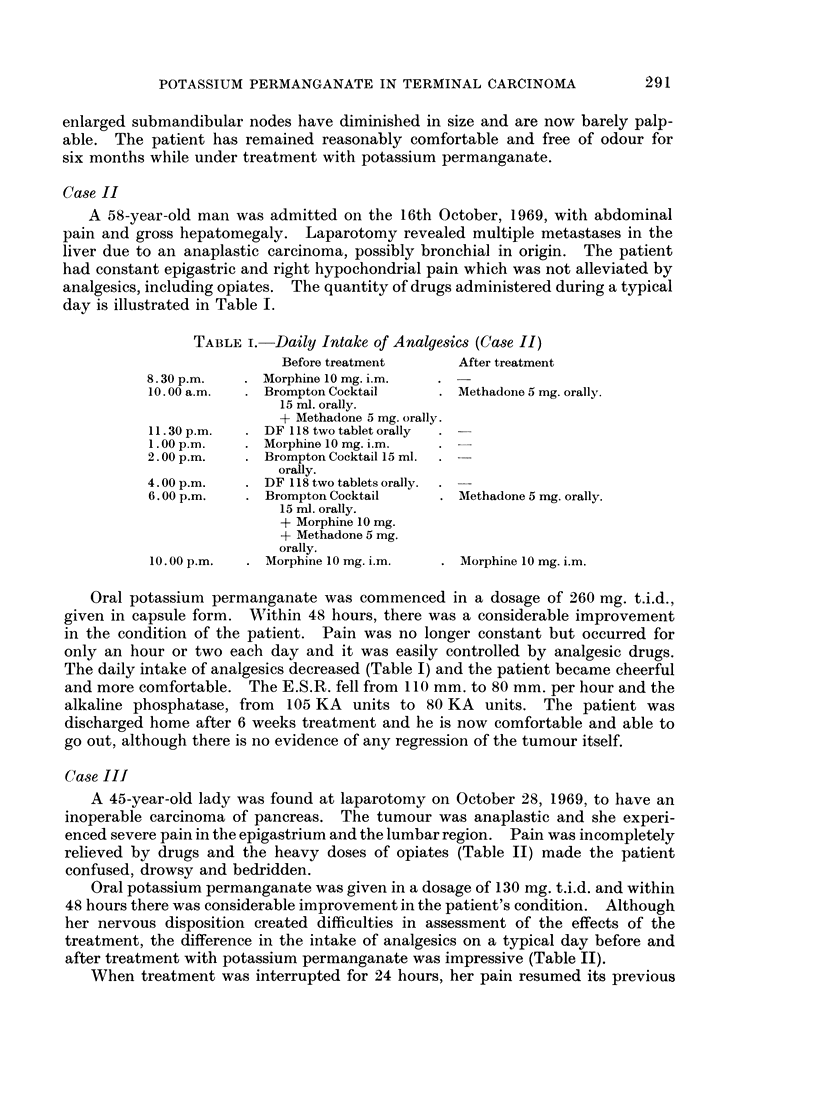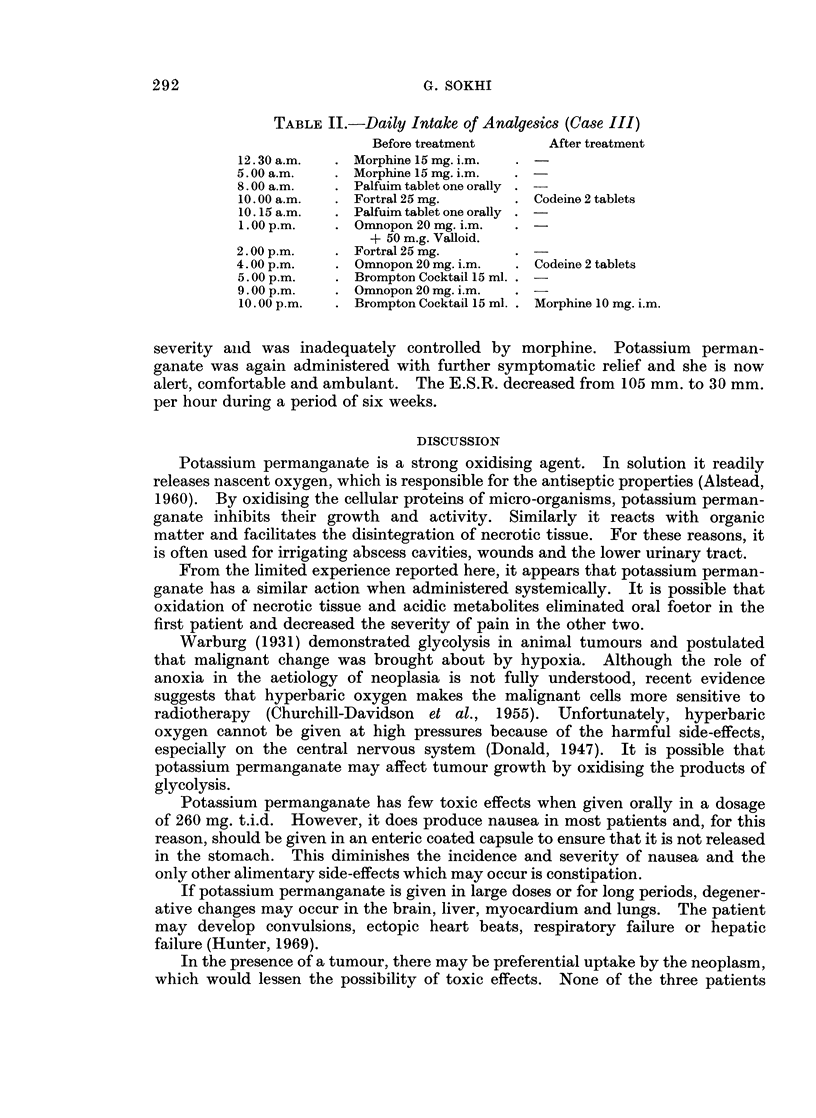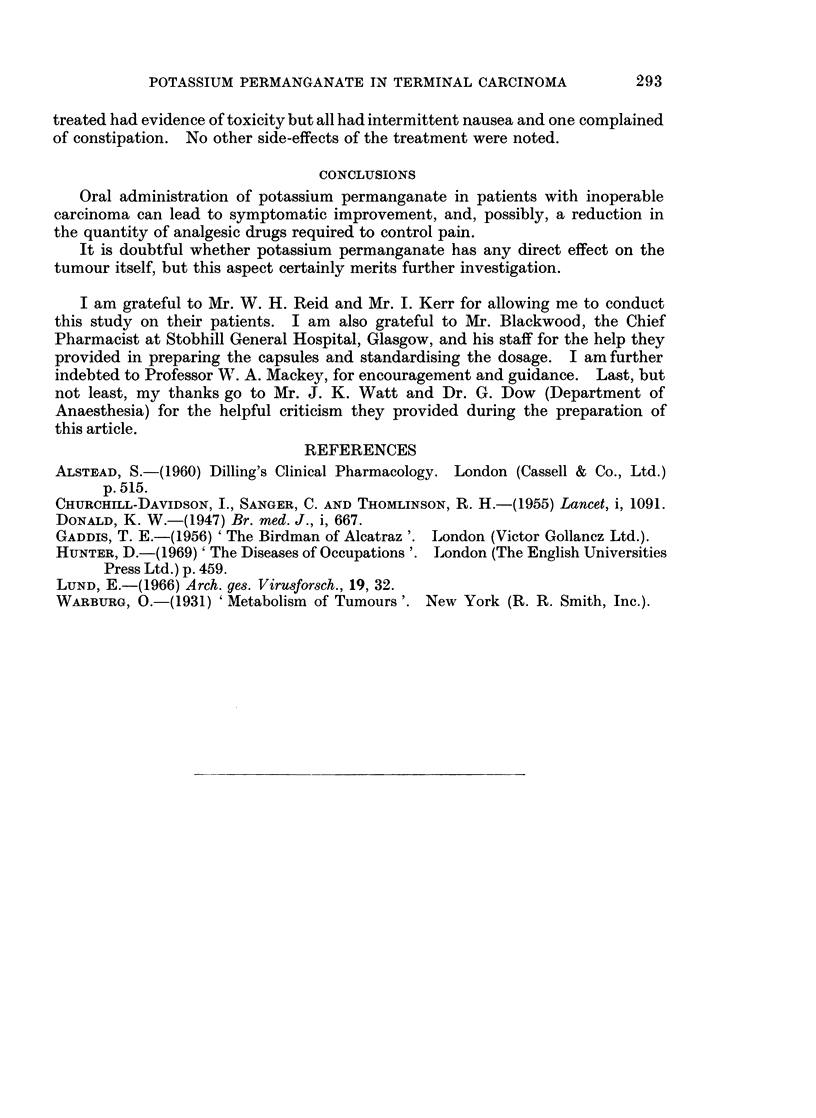# The Management of Terminal Carcinoma with Oral Potassium Permanganate

**DOI:** 10.1038/bjc.1970.33

**Published:** 1970-06

**Authors:** G. Sokhi

## Abstract

Oral potassium permanganate has been used in the management of terminal carcinoma in three patients. Symptomatic improvement occurred in all three, with elimination of oral foetor in one patient and diminished requirement of analgesics in the other two. The mental state of each patient was improved and normal activities were resumed.


### THE MANAGEMENT OF TERMINAL CARCINOMA WITH ORAL POTASSIUM PERMANGANATE

G. SOKHI

*From the Royal Infirmary, Glasgow*

Received for publication March 20, 1970

**SUMMARY.—Oral potassium permanganate has been used in the management of terminal carcinoma in three patients. Symptomatic improvement occurred in all three, with elimination of oral foetor in one patient and diminished requirement of analgesics in the other two. The mental state of each patient was improved and normal activities were resumed.**

STROUD, the " Birdman of Alcatraz ", demonstrated (Gaddis, 1956) that a suitable antiseptic used as a whole body detergent could enable the host to overcome the deleterious effects of a pathogen. By using oxidising agents he succeeded in eliminating viral infection from birds. There is now more recent evidence (Lund, 1966) that oxidising agents may also have a direct inactivating action on some viruses.

Potassium permanganate is not toxic to human beings when used in small doses, except when it is administered for very long periods. It has proved useful in the management of patients with carcinoma in whom other methods of treatment had been unable to give symptomatic relief. These patients were in the terminal stages of the disease and had such troublesome symptoms that life was absolutely miserable.

### CASE REPORTS

### *Case I*

A 71-year-old woman had a left-sided mandibulectomy in 1967 for squamous cell carcinoma of floor of mouth. The carcinoma recurred following a course of post-operative radiotherapy and she was readmitted in July 1969, with an inoperable lesion. There was infiltration of tongue, cervical lymph nodes and the contralateral mandibular region. A further short course of radiotherapy produced no improvement.

Her most troublesome symptom was a strong smell which caused distress not only to the patient and her relatives but also to the other occupants of the ward.

A capsule containing 260 mg. of crystalline potassium permanganate was given orally three times daily and within 48 hours the oral foetor was abolished and the patient became brighter and more active. Her appetite improved as well. After two months, treatment was discontinued for one week and the nauseating foetor recurred with all its distressing effects. Treatment was recommenced (in fact, it was demanded by her daughter) and her foetor was again controlled satisfactorily.

At the present time, there is still oedema of the tongue and a fistula through the lip, but the primary growth has become softer in consistency and the grossly enlarged submandibular nodes have diminished in size and are now barely palpable. The patient has remained reasonably comfortable and free of odour for six months while under treatment with potassium permanganate.

*Case II*

A 58-year-old man was admitted on the 16th October, 1969, with abdominal pain and gross hepatomegaly. Laparotomy revealed multiple metastases in the liver due to an anaplastic carcinoma, possibly bronchial in origin. The patient had constant epigastric and right hypochondrial pain which was not alleviated by analgesics, including opiates. The quantity of drugs administered during a typical day is illustrated in Table I.

TABLE I.—*Daily Intake of Analgesics* (*Case II*)

| | Before treatment | After treatment |
|---|---|---|
| 8.30 p.m. | Morphine 10 mg. i.m. | — |
| 10.00 a.m. | Brompton Cocktail 15 ml. orally. + Methadone 5 mg. orally. | Methadone 5 mg. orally. |
| 11.30 p.m. | DF 118 two tablet orally | — |
| 1.00 p.m. | Morphine 10 mg. i.m. | — |
| 2.00 p.m. | Brompton Cocktail 15 ml. orally. | — |
| 4.00 p.m. | DF 118 two tablets orally. | — |
| 6.00 p.m. | Brompton Cocktail 15 ml. orally. + Morphine 10 mg. + Methadone 5 mg. orally. | Methadone 5 mg. orally. |
| 10.00 p.m. | Morphine 10 mg. i.m. | Morphine 10 mg. i.m. |

Oral potassium permanganate was commenced in a dosage of 260 mg. t.i.d., given in capsule form. Within 48 hours, there was a considerable improvement in the condition of the patient. Pain was no longer constant but occurred for only an hour or two each day and it was easily controlled by analgesic drugs. The daily intake of analgesics decreased (Table I) and the patient became cheerful and more comfortable. The E.S.R. fell from 110 mm. to 80 mm. per hour and the alkaline phosphatase, from 105 KA units to 80 KA units. The patient was discharged home after 6 weeks treatment and he is now comfortable and able to go out, although there is no evidence of any regression of the tumour itself.

*Case III*

A 45-year-old lady was found at laparotomy on October 28, 1969, to have an inoperable carcinoma of pancreas. The tumour was anaplastic and she experienced severe pain in the epigastrium and the lumbar region. Pain was incompletely relieved by drugs and the heavy doses of opiates (Table II) made the patient confused, drowsy and bedridden.

Oral potassium permanganate was given in a dosage of 130 mg. t.i.d. and within 48 hours there was considerable improvement in the patient's condition. Although her nervous disposition created difficulties in assessment of the effects of the treatment, the difference in the intake of analgesics on a typical day before and after treatment with potassium permanganate was impressive (Table II).

When treatment was interrupted for 24 hours, her pain resumed its previous

TABLE II.—*Daily Intake of Analgesics (Case III)*

| | Before treatment | After treatment |
|---|---|---|
| 12.30 a.m. | Morphine 15 mg. i.m. | — |
| 5.00 a.m. | Morphine 15 mg. i.m. | — |
| 8.00 a.m. | Palfuim tablet one orally | — |
| 10.00 a.m. | Fortral 25 mg. | Codeine 2 tablets |
| 10.15 a.m. | Palfuim tablet one orally | — |
| 1.00 p.m. | Omnopon 20 mg. i.m. + 50 m.g. Valloid. | — |
| 2.00 p.m. | Fortral 25 mg. | — |
| 4.00 p.m. | Omnopon 20 mg. i.m. | Codeine 2 tablets |
| 5.00 p.m. | Brompton Cocktail 15 ml. | — |
| 9.00 p.m. | Omnopon 20 mg. i.m. | — |
| 10.00 p.m. | Brompton Cocktail 15 ml. | Morphine 10 mg. i.m. |

severity and was inadequately controlled by morphine. Potassium permanganate was again administered with further symptomatic relief and she is now alert, comfortable and ambulant. The E.S.R. decreased from 105 mm. to 30 mm. per hour during a period of six weeks.

### DISCUSSION

Potassium permanganate is a strong oxidising agent. In solution it readily releases nascent oxygen, which is responsible for the antiseptic properties (Alstead, 1960). By oxidising the cellular proteins of micro-organisms, potassium permanganate inhibits their growth and activity. Similarly it reacts with organic matter and facilitates the disintegration of necrotic tissue. For these reasons, it is often used for irrigating abscess cavities, wounds and the lower urinary tract.

From the limited experience reported here, it appears that potassium permanganate has a similar action when administered systemically. It is possible that oxidation of necrotic tissue and acidic metabolites eliminated oral foetor in the first patient and decreased the severity of pain in the other two.

Warburg (1931) demonstrated glycolysis in animal tumours and postulated that malignant change was brought about by hypoxia. Although the role of anoxia in the aetiology of neoplasia is not fully understood, recent evidence suggests that hyperbaric oxygen makes the malignant cells more sensitive to radiotherapy (Churchill-Davidson *et al.*, 1955). Unfortunately, hyperbaric oxygen cannot be given at high pressures because of the harmful side-effects, especially on the central nervous system (Donald, 1947). It is possible that potassium permanganate may affect tumour growth by oxidising the products of glycolysis.

Potassium permanganate has few toxic effects when given orally in a dosage of 260 mg. t.i.d. However, it does produce nausea in most patients and, for this reason, should be given in an enteric coated capsule to ensure that it is not released in the stomach. This diminishes the incidence and severity of nausea and the only other alimentary side-effects which may occur is constipation.

If potassium permanganate is given in large doses or for long periods, degenerative changes may occur in the brain, liver, myocardium and lungs. The patient may develop convulsions, ectopic heart beats, respiratory failure or hepatic failure (Hunter, 1969).

In the presence of a tumour, there may be preferential uptake by the neoplasm, which would lessen the possibility of toxic effects. None of the three patients treated had evidence of toxicity but all had intermittent nausea and one complained of constipation. No other side-effects of the treatment were noted.

### CONCLUSIONS

Oral administration of potassium permanganate in patients with inoperable carcinoma can lead to symptomatic improvement, and, possibly, a reduction in the quantity of analgesic drugs required to control pain.

It is doubtful whether potassium permanganate has any direct effect on the tumour itself, but this aspect certainly merits further investigation.

I am grateful to Mr. W. H. Reid and Mr. I. Kerr for allowing me to conduct this study on their patients. I am also grateful to Mr. Blackwood, the Chief Pharmacist at Stobhill General Hospital, Glasgow, and his staff for the help they provided in preparing the capsules and standardising the dosage. I am further indebted to Professor W. A. Mackey, for encouragement and guidance. Last, but not least, my thanks go to Mr. J. K. Watt and Dr. G. Dow (Department of Anaesthesia) for the helpful criticism they provided during the preparation of this article.

### REFERENCES

ALSTEAD, S.—(1960) Dilling's Clinical Pharmacology. London (Cassell & Co., Ltd.) p. 515.

CHURCHILL-DAVIDSON, I., SANGER, C. AND THOMLINSON, R. H.—(1955) *Lancet*, i, 1091.

DONALD, K. W.—(1947) *Br. med. J.*, i, 667.

GADDIS, T. E.—(1956) ‘The Birdman of Alcatraz’. London (Victor Gollancz Ltd.).

HUNTER, D.—(1969) ‘The Diseases of Occupations’. London (The English Universities Press Ltd.) p. 459.

LUND, E.—(1966) *Arch. ges. Virusforsch.*, **19**, 32.

WARBURG, O.—(1931) ‘Metabolism of Tumours’. New York (R. R. Smith, Inc.).